# Examination of metal mobilization from a gunshot by scanning acoustic microscopy, scanning electron microscopy, energy-dispersive X-ray spectroscopy, and inductively coupled plasma optical emission spectroscopy: a case report

**DOI:** 10.1186/s13256-018-1905-7

**Published:** 2018-12-28

**Authors:** Bukem Bilen, Fatma Ates Alkan, Umit Bora Barutcu, Meltem Sezen, Mehmet Burcin Unlu, Kamran Aghayev

**Affiliations:** 1 Physics Department, Bogazici University, Istanbul, Turkey; 2 Biophysics Department, Faculty of Medicine, Beykent University, Istanbul, Turkey; 3 Biophysics Department, Cerrahpasa Faculty of Medicine, Istanbul University, Istanbul, Turkey; 4 Sabanci University Nanotechnology Research and Application Center, Sabanci University, Istanbul, Turkey; 5 Biruni University Hospital, Istanbul, Turkey

**Keywords:** Metal infiltration, Lead toxicity, Scanning acoustic microscopy, Gunshot injury

## Abstract

**Background:**

Projectile foreign bodies are known to cause chronic heavy metal toxicity due to the release of metal into the bloodstream. However, the local effect around the metallic object has not been investigated and the main goal of our study is to examine the influence of the object in close proximity of the object.

**Case presentation:**

A 36-year-old Caucasian woman with one metallic pellet close to her sciatic nerve due to a previous shotgun injury at the gluteal area presented with a diagnosis of recurrent lumbar disk herniation at L4–5 level. A physical examination confirmed chronic neuropathy and she underwent a two-stage surgery. The surgery included removal of the foreign body, followed by discectomy and fusion at the involved level. During the removal of the metallic foreign body, a tissue sample around the pellet and another tissue sample from a remote area were obtained. The samples were analyzed by scanning acoustic microscopy, scanning electron microscopy, and energy-dispersive X-ray spectroscopy. Lead, chromium, copper, cadmium, iron, manganese, selenium, and zinc elements in tissue, blood, and serum specimens were detected by inductively coupled plasma optical emission spectroscopy.

**Conclusions:**

An acoustic impedance map of the tissue closer to the metallic body showed higher values indicating further accumulation of elements. Energy-dispersive X-ray spectroscopy results confirmed scanning acoustic microscopy results by measuring a higher concentration of elements closer to the metallic body. Scanning electron microscopy images showed that original structure was not disturbed far away; however, deformation of the structure existed in the tissue closer to the foreign body. Element analysis showed that element levels within blood and serum were more or less within acceptable ranges; on the other hand, element levels within the tissues showed pronounced differences indicating primarily lead intoxication in the proximity of the metallic body. We can state that residues of metallic foreign bodies of gunshot injuries cause chronic metal infiltration to the surrounding tissue and induce significant damage to nearby neural elements; this is supported by the results of scanning acoustic microscopy, scanning electron microscopy, energy-dispersive X-ray spectroscopy, and inductively coupled plasma optical emission spectroscopy.

## Background

Metallic foreign bodies from gunshot injuries are typical in clinical practice. Usually, they require no treatment. However, lead (Pb) release from retained bullets may cause systemic toxicity [1–6]. It is generally accepted that foreign particles embedded in soft tissues become encapsulated and induce no harm to the patient. On the other hand, at specific locations, such as joints, bullets or fragments cause subsequent Pb wash out and systemic toxicity [7, 8]. Pb toxicity, caused by these gunshot fragments, has been discussed for many years [4, 9, 10]; however, there is no agreement on the immediate removal of the fragments from the body [11–15]. Even though blood levels are not routinely monitored, a few studies showed increased blood Pb levels [3, 7, 15, 16]. It was also reported that increased Pb concentration provoked a reduction in iron (Fe) and calcium (Ca) concentrations altering the ratios of Fe/copper (Cu), Fe/zinc (Zn), and Ca/Zn not only in blood, but also in hair [17].

Scanning acoustic microscopy (SAM) is a modality capable of obtaining information about the structural and elastic properties of biological samples at microscopic levels. With high-frequency ultrasound signals and without using any stains for the specimens, two-dimensional maps can be obtained within minutes. SAM has two modes of measurement: speed of sound (SOS) and acoustic impedance (AI). SOS mode calculates the sound speed passing through the tissues [18–27], while AI mode calculates the AI of samples [28, 29]. Use of higher frequencies of 100 to 1200 MHz within this microscope enables resolving cells and organelles [30–37]. Scanning electron microscopy (SEM) is another imaging technique used for investigating specimen surfaces at very high magnifications and even higher resolutions. A focused beam of electrons scans the material surface resulting in signal generations due to beam-sample interaction, which in turn can be collected as images with different contrast mechanisms. Energy-dispersive X-ray spectroscopy (EDS) enables the detection of elements and their distribution within samples. A bombardment of the specimen surface with a focused electron beam causes characteristic X-ray spectrum emission, which then can be used to obtain a local chemical analysis. In principle, using EDS measurements, all elements ranging from beryllium (Be) to uranium (U) can be detected. EDS can be performed in SEM or transmission electron microscopy (TEM) systems. Gunshot residue analyses are done successfully by SEM equipped with EDS [38, 39].

The majority of scientific papers related to the long-term effect of gun injuries are focused on systemic effects. However, there is evidence that foreign bodies may cause local effects and damage to anatomical structures in close proximity. This local effect has not been studied in detail so far, and, in this research, we aimed to study the local influence of a bullet close to a sciatic nerve by SAM, SEM, EDS, and inductively coupled plasma optical emission spectroscopy (ICP-OES).

## Case presentation

### Surgical technique and sampling

**Fig. 1:**
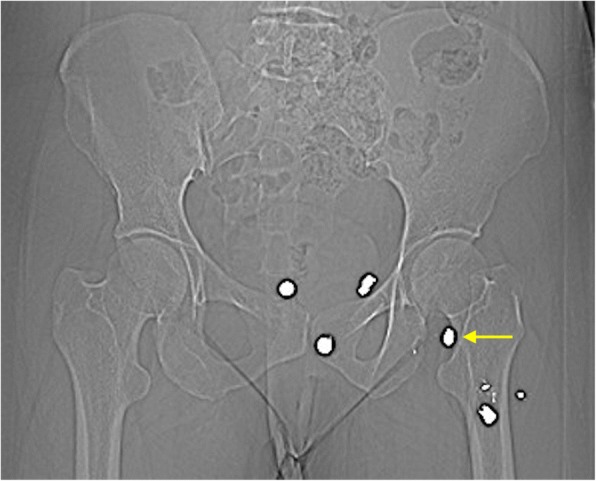
Preoperative computed tomography scan demonstrating multiple shotgun pellets in the pelvis and upper thigh area. Pellet of interest is marked with *yellow arrow*

**Fig. 2:**
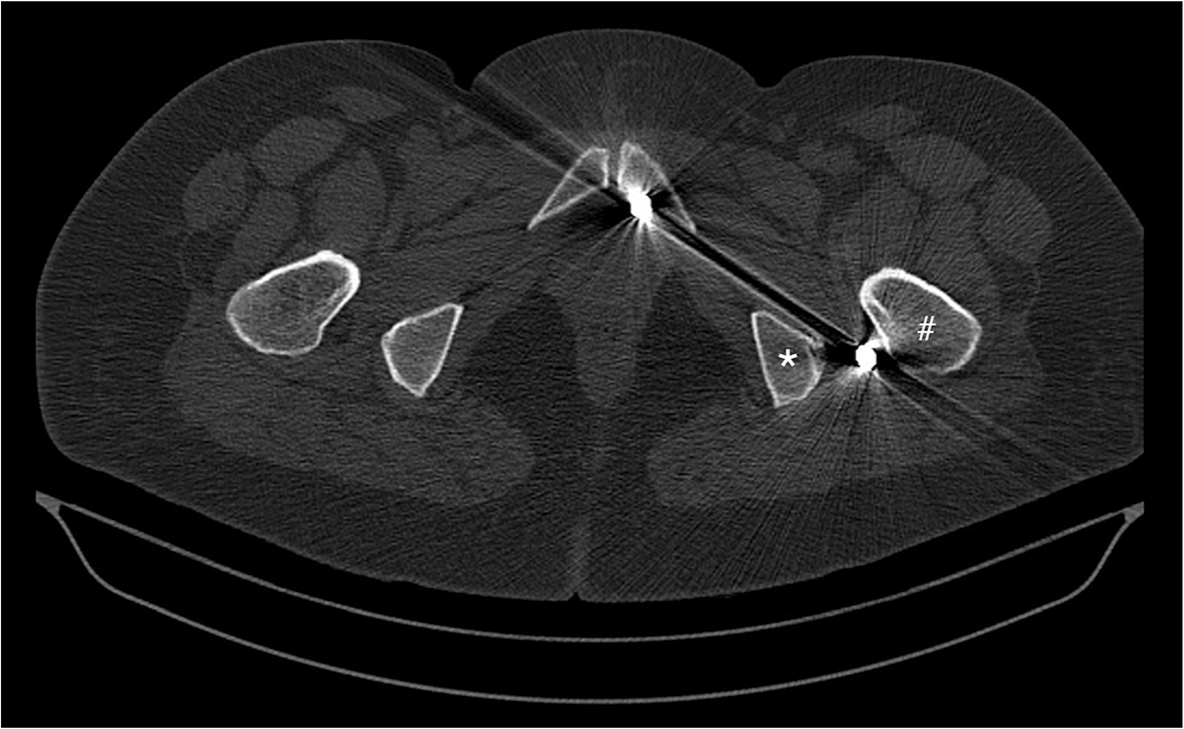
Preoperative computed tomography scan demonstrating the pellet of interest located between the trochanter major (#) and ischial tuberosity (*) close to the course of sciatic nerve

A 36-year-old Caucasian woman was evaluated with chief complaint of gluteal pain radiating to her leg. Her medical history was remarkable with gunshot injury to the affected leg with multiple pellets dispersed into her pelvis and proximal part of the thigh, as shown in Figs. 1 and 2. She had gunshot injury 20 years ago. She was previously diagnosed as having lumbar disc herniation at L4–5 level. She underwent a previous discectomy outside our institution 2 years ago. A radiological examination revealed the presence of recurrent disc herniation, as well as multiple shotgun bullets in her pelvis and thigh. One of those bullets was deep into the sciatic nerve inside her quadratus femoris muscle.

Electromyography (EMG) showed the presence of chronic sciatic nerve injury. Since it was clinically impossible to distinguish lumbar disc herniation from the sciatic injury, we decided to proceed with removal of the foreign object and neurolysis of the sciatic nerve followed by L4–5 discectomy and fusion. We decided to perform those procedures in two different settings. The first surgery included access to the sciatic nerve in the upper portion of her thigh and exposing the nerve fibrotic bands around the nerve. The dissection proceeded deep into the nerve within a muscle, where a bullet was found and removed. The distance from the bullets to the nerve was approximately 2 cm. Muscle tissue around the bullets was excised for analysis. For comparison, another specimen was obtained from the gluteal muscle, superficially away from the nerve and all the bullets. Two weeks later, she underwent scheduled L4–5 discectomy and fusion. Her postoperative course was uneventful. On follow-up examination at 6 months, she was essentially symptom free.

### SAM

**Fig. 3:**
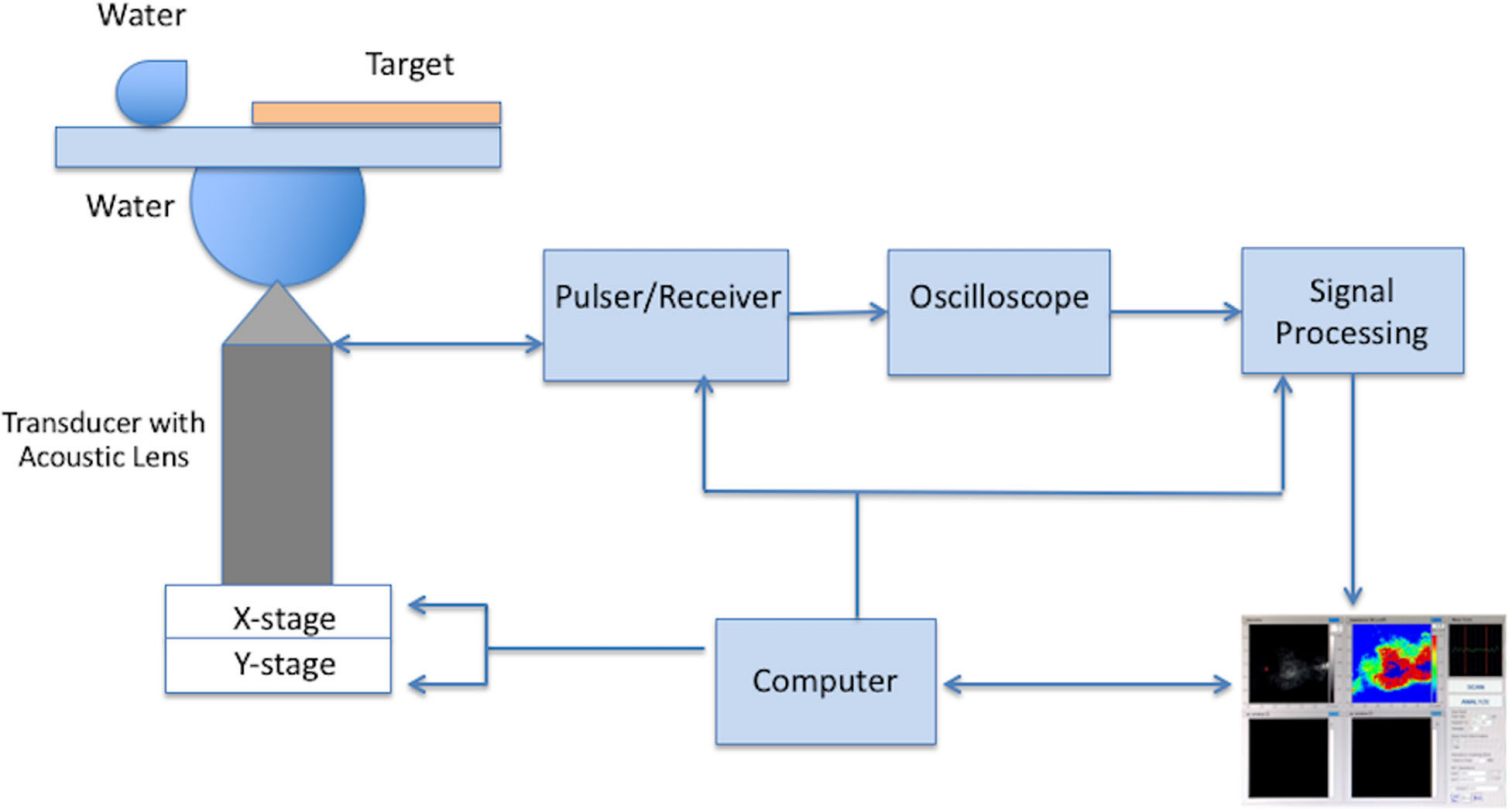
Schematic of scanning acoustic microscopy setup operated in acoustic impedance mode

A scanning acoustic microscope (AMS-50SI) developed by Honda Electronics (Toyohashi, Japan), whose schematic setup is shown in Fig. 3, was used in AI mode. It has a transducer with quartz lens, a pulser/receiver, an oscilloscope, a computer, and a display monitor. An 80 MHz transducer is installed within the microscope, which generates the signals and collects the reflected acoustic waves. Water is the coupling medium between the quartz lens and the substrate. For two-dimensional scans, an X-Y stage, controlled by a computer, is used. An oscilloscope analyzes the reflected signals from both the reference and target material after being collected by the transducer. As a result, acoustic intensity and impedance maps of the region of interest with 300 × 300 sampling points are obtained.

**Fig. 4:**
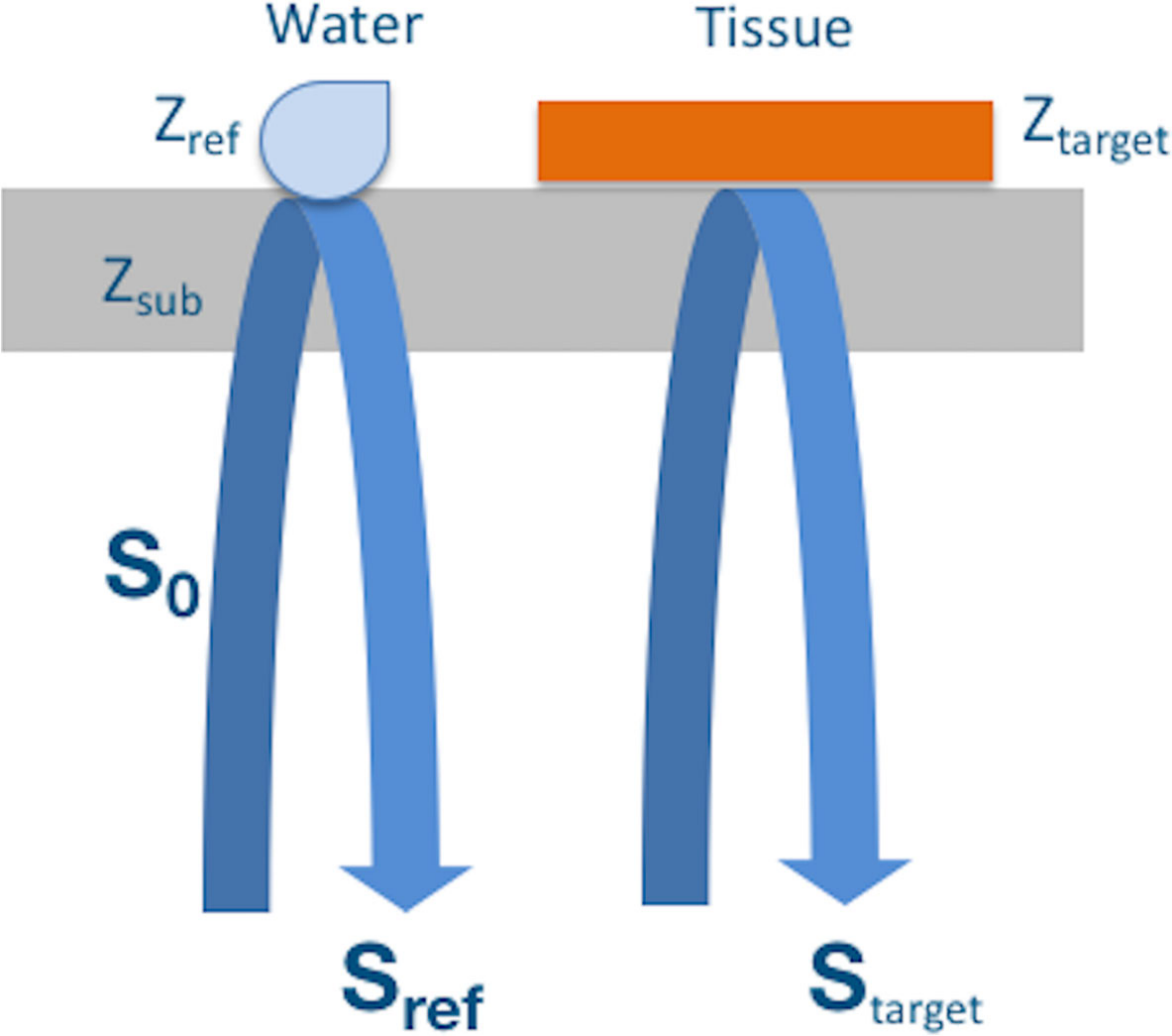
Principle of scanning acoustic microscopy in acoustic impedance mode. The acoustic waves reflected from the surfaces of water and the tissue are collected by the same transducer and then combined for the calculation of the acoustic impedance of the tissue. *S*_0_ is the generated signal by the 80 MHz transducer, *Z*_ref_ is the acoustic impedance of water, *Z*_sub_ is the polystyrene substrate’s acoustic impedance, *Z*_target_ is tissue’s acoustic impedance

The principle of SAM in AI mode is demonstrated in Fig. 4. Distilled water is widely used as reference. The signal reflected from the target is

1 $$ \kern0.50em {S}_{target}=\frac{Z_{target}-{Z}_{sub}}{Z_{target}+{Z}_{sub}}{S}_0 $$

where, *S*_0_ is the generated signal by the 80 MHz transducer, *Z*_target_ is tissue’s AI and *Z*_sub_ is the polystyrene substrate’s AI (2.37 MRayl). The tissue’s AI is calculated by combining the reflected signals from the tissue and the reference. The signal reflected from the reference is

2 $$ {S}_{ref}=\frac{Z_{\mathrm{r} ef}-{Z}_{sub}}{Z_{ref}+{Z}_{sub}}{S}_0 $$

where *Z*_ref_ is the AI of water (1.50 MRayl). Then, the target’s AI is written as

3 $$ {Z}_{target}=\frac{1+\frac{S_{target}}{S_0}}{1-\frac{S_{target}}{S_0}}{Z}_{sub} $$

with a constant signal *S*_0_ [28] generated by the transducer.

### SEM and EDS

**Fig. 5:**
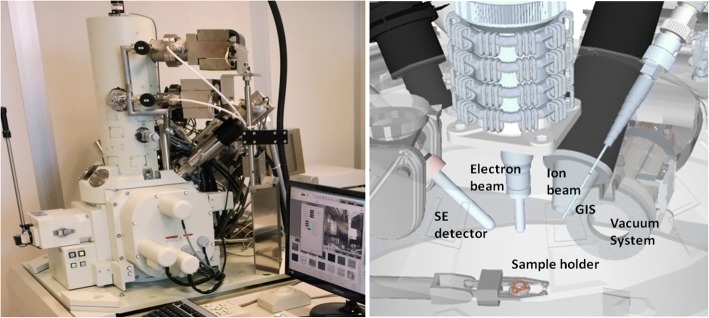
JEOL JIB-4601 focused ion beam scanning electron microscope multi-beam platform coupled with Oxford X-MaxN energy-dispersive X-ray spectroscopy system. *GIS* gas injection system, *SE* secondary electron

Electron microscopy-based imaging and chemical analysis studies were performed in a JEOL JIB-4601 focused ion beam scanning electron microscope (FIB-SEM) multi-beam platform coupled with an Oxford X-MaxN EDS system, as shown in Fig. 5.

### Element analysis

Blood samples were collected in test tubes containing ethylenediamine-tetraacetic acid (EDTA) and no anticoagulant on the day of the first surgery (foreign object removal) prior to the procedure. Then, 2 ml of 20% trichloroacetic acid (TCA) was supplemented into the blood samples to release the red blood cells (RBC) and other ingredients. The supernatant part was received from blood with TCA by centrifugation at 4000 revolutions per minute (rpm) for 20 minutes for the analysis of Pb and cadmium (Cd) within total blood. Coagulation of blood samples enabled serum trace element analysis: chromium (Cr), Fe, Cu, magnesium (Mg), manganese (Mn), selenium (Se), and Zn. The serum specimen was prepared using Hettich Universal centrifuge by centrifugation at 3000 rpm for 15 minutes, separating from cells immediately after and storing at − 20 °C until the analysis [40].

After weighing the left sciatic nerve tissue samples, they were digested with 2 ml of 65% nitric acid (HNO_3_) at 180 °C in the incubator for 1 hour. Then, 2 ml of 65% perchloric acid (HClO_4_) was added into the cooled mixture. Then, the mixture was digested at 200 °C in the incubator until the volume was halved. Digested materials were vortexed and diluted in water to a total volume of 10 ml. Concentrations were given in micrograms per gram (μg/g) wet tissue weight [41].

All glassware were maintained at 10% (volume/volume; v/v) HNO_3_ before use, cleaned with deionized water, and dried in an incubator at 100 °C overnight. Pb, Cd, Cu, Cr, Fe, Mn, Se, and Zn elements were detected by inductively coupled plasma optical emission spectrophotometer (ICP-OES 6000, Thermo, Cambridge, United Kingdom). Measurements for each element were done three times and averaged. The ICP-OES was operated with argon carrier flow rate of 0.5 L/minute, plasma gas flow rate of 15 L/minute, sample flow and elusion rate of 1.51 L/minute, and peristaltic pump speed of 100 rpm, selecting the suitable wavelength for Pb, Cd, Cr, Cu, Fe, Mn, Se, and Zn, which were 220.353 nm, 228.802 nm, 267.716 nm, 324.75 nm, 285.213 nm, 357.610 nm, 196.090 nm, and 206.200 nm, respectively. Transport lines were obtained using 1.25 mm internal diameter polytetrafluoroethylene tubing. Element levels were indicated in micrograms per deciliter for serum (μg/dl) and μg/g for wet tissue. The standard concentrations for standard graph calibration were arranged from standard stock solutions of 1000 μg/ml for each analyzed element [42].

### SAM results

**Fig. 6:**
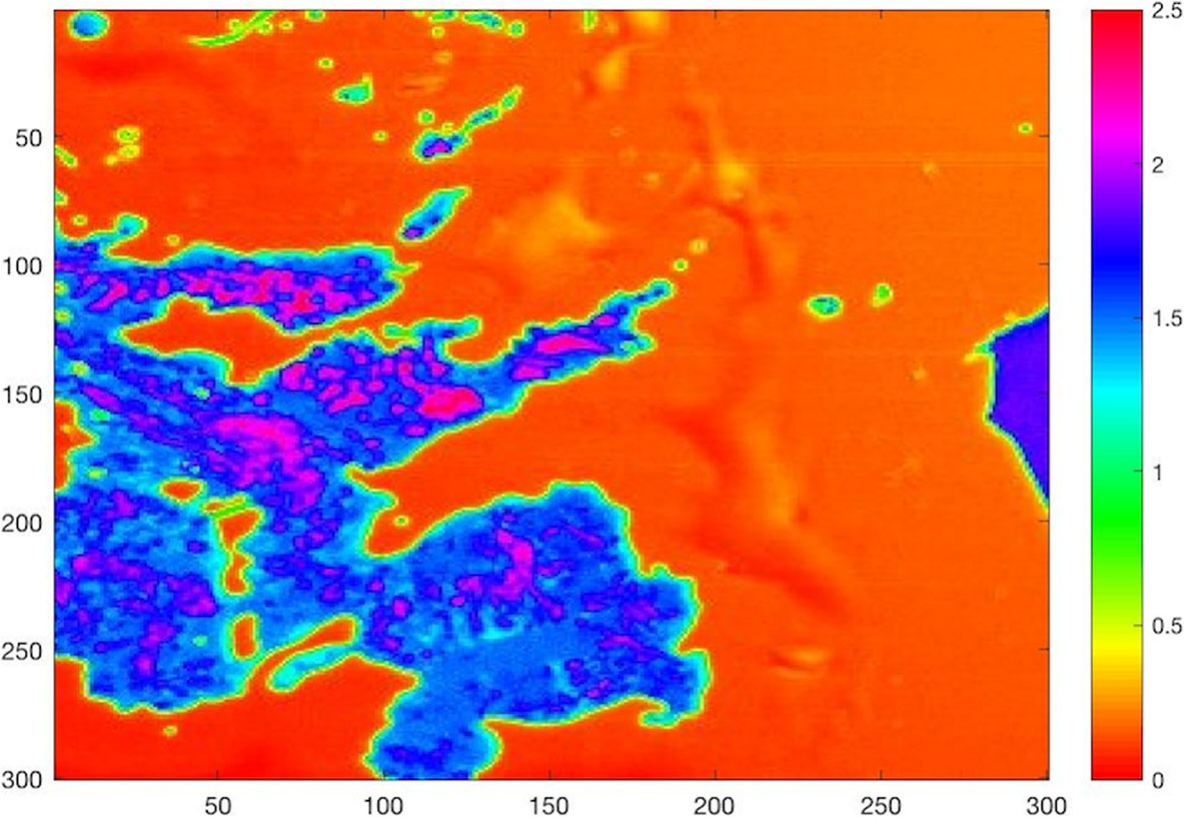
Acoustic impedance map of the tissue obtained away from the gunshot by comparing the reflected ultrasound signals from the surfaces of water and the sample

**Fig. 7:**
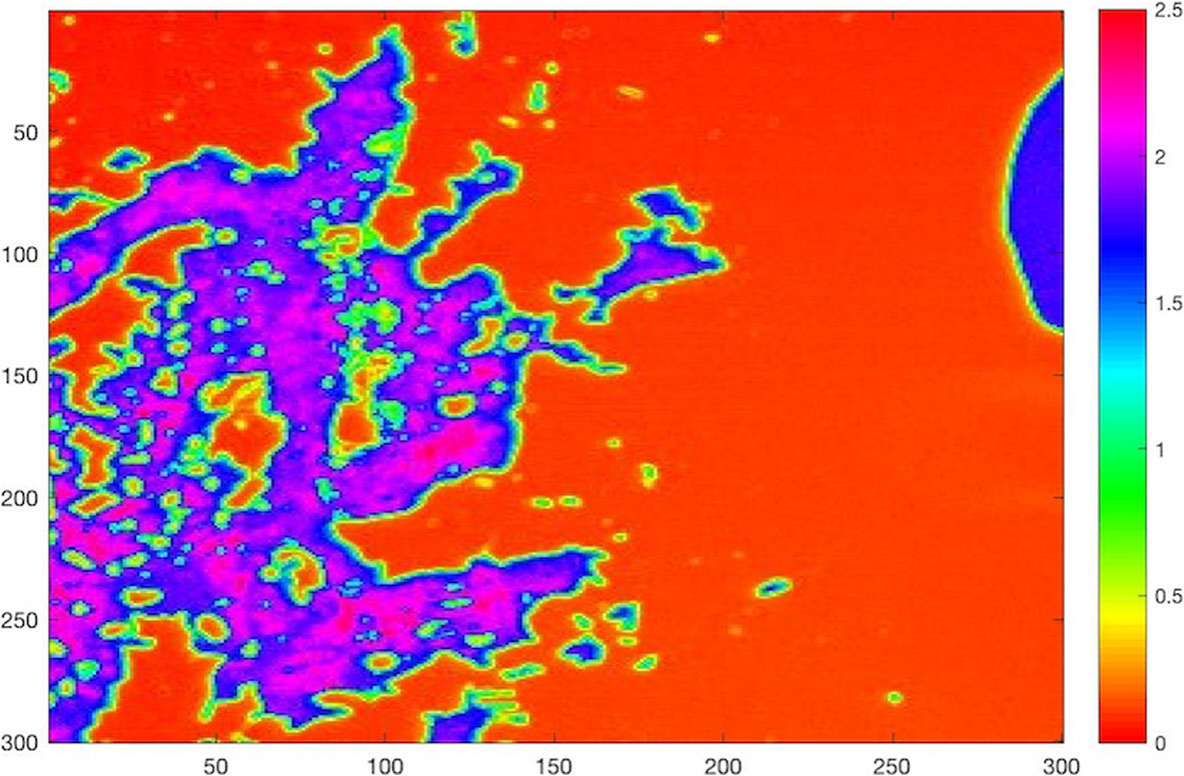
Acoustic impedance map of the tissue obtained close to the gunshot by comparing the reflected ultrasound signals from the surfaces of water and the sample

The tissue samples were investigated by using AI mode of SAM. Figure 6 shows the AI map of the tissue obtained away from the gunshot. The map was constructed by collecting the reflections of acoustic signals, generated by the transducer within SAM, from surfaces of the reference (water) and the tissue sample on the polystyrene substrate. At specific locations within the sample, the AI was calculated to be higher than 2 MRayl, indicating accumulation of elements with different elastic properties. Figure 7 shows the AI map of the tissue obtained close to the gunshot. As can be seen in this image, almost everywhere had an AI of greater than 2 MRayl.

### SEM and EDS results

**Fig. 8:**
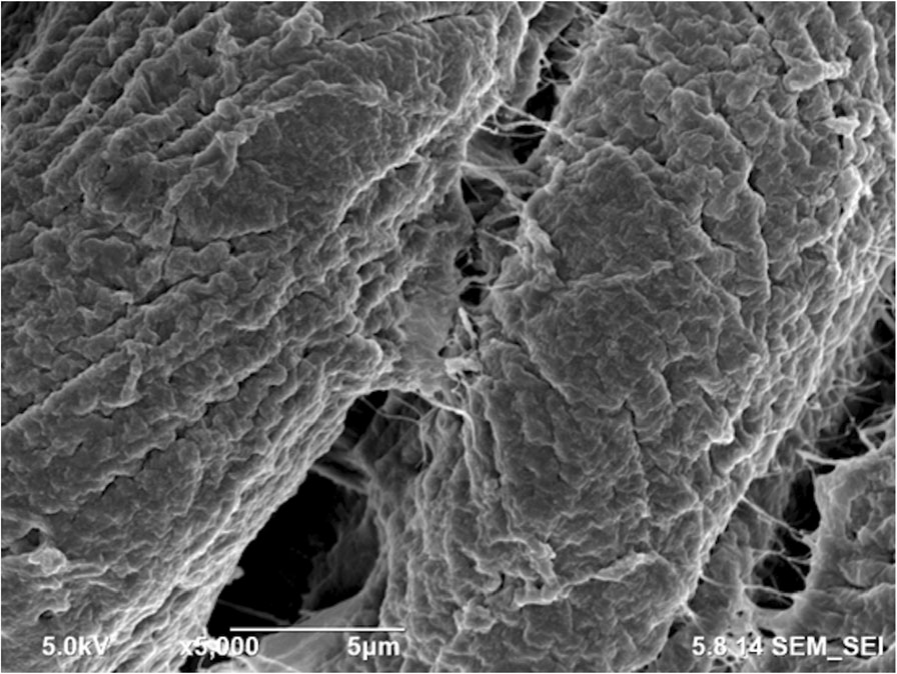
Scanning electron microscopy image of the tissue obtained away from the gunshot with 5000 × magnification. The tissue seems more or less intact since less damage occurred in this region

**Fig. 9:**
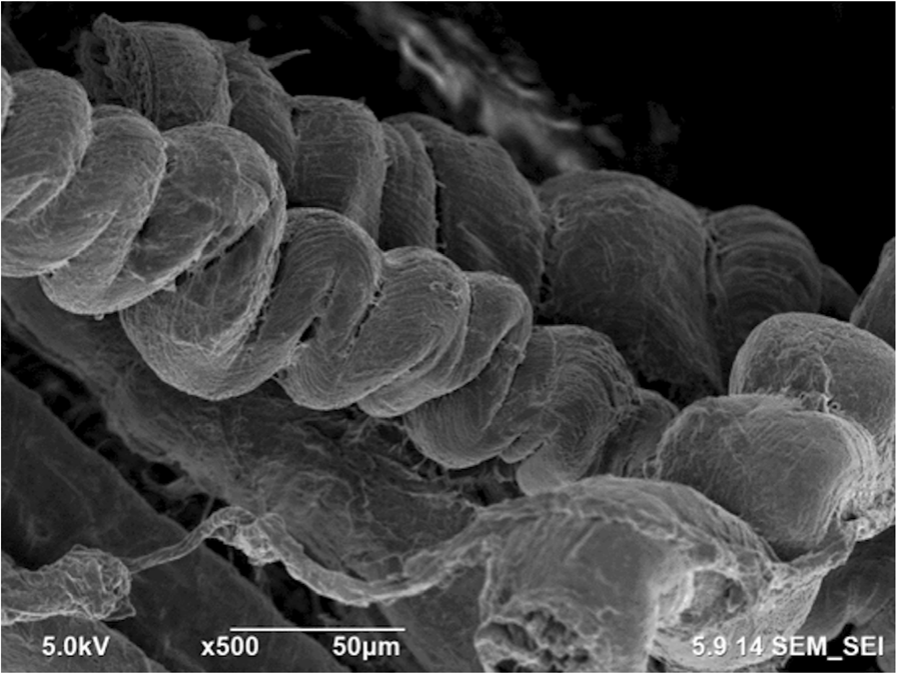
Scanning electron microscopy image of the tissue obtained away from the gunshot with 500 × magnification. The tissue keeps its original structure with aligned tendons

**Fig. 10:**
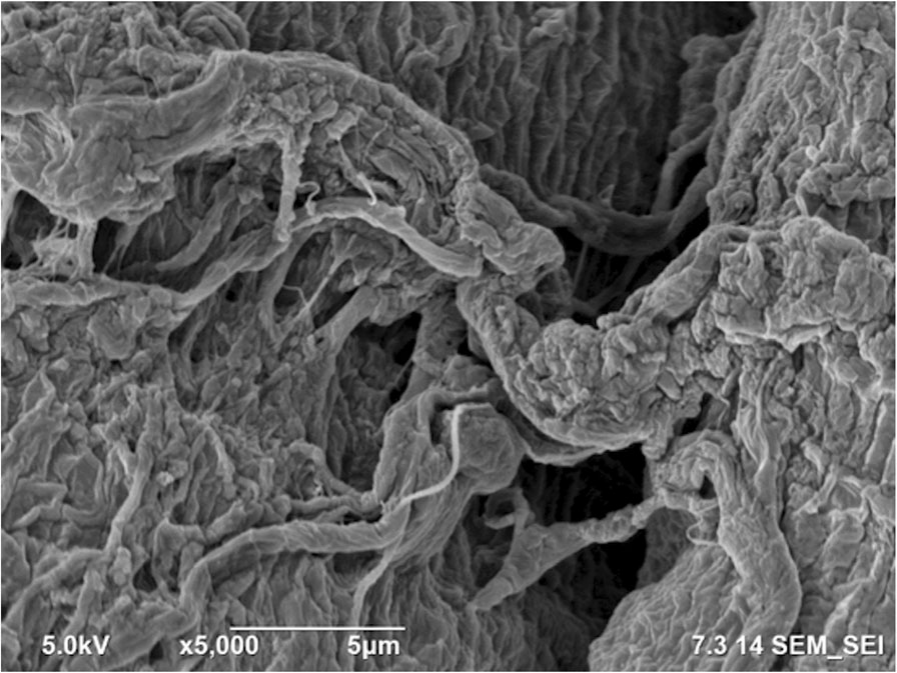
Scanning electron microscopy image of the tissue obtained close to the gunshot with 5000 × magnification. Due to the damage caused by gunshot impact, the tissue seems torn

**Fig. 11:**
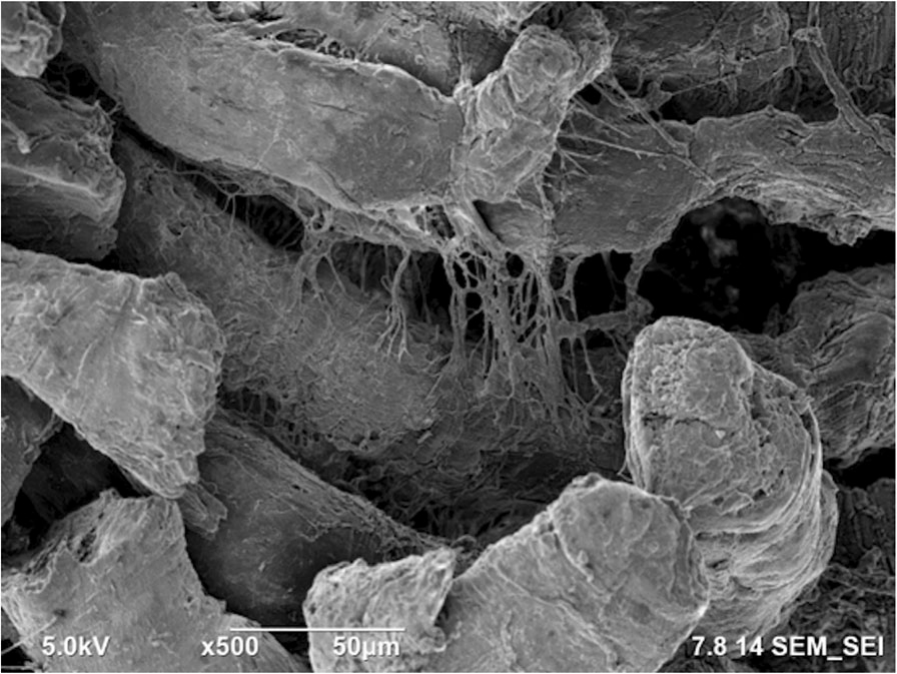
Scanning electron microscopy image of the tissue obtained close to the gunshot with 500 × magnification. Tendons are not in the form of straight arrays

SEM images of the tissue far away from the gunshot were obtained at magnifications of 5000 × and 500 ×, as shown in Figs. 8 and 9, respectively. Similarly, SEM images for the tissue close to the gunshot were obtained at magnifications of 5000 × and 500 ×, as shown in Figs. 10 and 11, respectively. The images were acquired at 5 keV energy for both tissue samples.

The SEM images show that the tissue far away from the gunshot keeps its original structure, whereas the tissue close to the gunshot seems to be deformed and torn up. These results demonstrate the degree of damage the impact of gunshot causes on soft biological tissues.

Table 1 represents the EDS measurements in SEM, carried out for determining the elemental distribution differences in the deformed tissues. The measurements show the percentages of the residue elements detected on tissues far away from the gunshot and close to the gunshot. According to the results, among all residue elements, Pb, Cr, Fe, and Mn are found to be higher in weight content in the region close to the gunshot, when compared to distant region. Cd and Cu levels do not differ much; however, Zn level is lower in the tissue close to the gunshot.

**Table 1:** Energy-dispersive X-ray spectroscopy analysis results. The measurements show the weight percentages of the residue elements within the tissues

Tissue	Cr	Mn	Fe	Cu	Zn	Cd	Pb
Close to the gunshot	0.54	1.15	1.72	10.70	41.46	1.05	42.22
Away from the gunshot	0.00	0.45	0.85	11.95	45.21	3.22	36.13

*Cd* cadmium, *Cr* chromium, *Cu* copper, *Fe* iron, *Mn* manganese, *Pb* lead, *Zn* zinc

### Element analysis results

We determined Pb, Cr, Cd, Cu, Fe, Mn, Zn, and Se levels in both tissue samples. Pb, Cr, Fe, Se, and Mn levels were higher in the tissue close to the gunshot, conversely, Zn level was lower in this sample (Table 2). Blood Pb and blood Cd, and serum Cr, Cu, Fe, Mn, Se, and Zn levels of our patient were also analyzed and the results are shown in Table 3, however, we did not observe significant differences when compared to reference values.

**Table 2:** Element levels within the tissues

Element	Tissue close to the gunshot	Tissue away from the gunshot
Pb (μg/g)	1.4716	0.0820
Cd (μg/g)	0.0355	0.0365
Cr (μg/g)	1.613	0.0520
Cu (μg/g)	1.418	1.281
Fe (μg/g)	1462.057	53.829
Mn (μg/g)	2.677	0.205
Se (μg/g)	0.816	0.178
Zn (μg/g)	33.440	39.011

*Cd* cadmium, *Cr* chromium, *Cu* copper, *Fe* iron, *Mn* manganese, *Pb* lead, *Se* selenium, *Zn* zinc

**Table 3:** Element levels within blood and serum

Element	Results	References
Blood Pb (μg/dl)	4.16	0–40
Blood Cd (μg/dl)	0.04	0–10
Serum Cr (μg/dl)	0.40	5–50
Serum Cu (μg/dl)	126.25	70–140
Serum Fe (μg/dl)	44.70	80–180
Serum Mn (μg/dl)	0.15	0.04–0.35
Serum Se (μg/dl)	14.75	7–13
Serum Zn (μg/dl)	27.95	70–120

*Cd* cadmium, *Cr* chromium, *Cu* copper, *Fe* iron, *Mn* manganese, *Pb* lead, *Se* selenium, *Zn* zinc

## Discussion

Foreign bodies such as bullets, shotgun pellets, and shrapnel can cause clinical symptoms by mechanical compression [43], lumen obstruction [44], irritation of nearby structures [45], systemic heavy metal intoxication [5], or tumor formation [46–49]. The first report of the systemic toxicity of the retained bullet dates back to the eighteenth century. Usually, metallic objects embedded into the soft tissue become encapsulated and do not release metals into systemic circulation. However, there are exceptions to this rule [45]. Missiles close to bone, especially if a fracture is present, are prone to systemic toxicity [50]. It is thought that initial impact with bone, fragments the missile with subsequent release [7]. Missiles lodged in or close to joints and intervertebral disks are continuously bathed with synovial fluid, which eventually washes off Pb from the bullets resulting in systemic toxicity [51–55]. Females are especially vulnerable to this form of toxicity [56]. It is logical to assume that the anatomical structures in close proximity to the retained bullets will suffer the most. For example, one study has demonstrated that bone fracture healing is impaired if there is a foreign metallic bullet [57]. Local metallosis is a well-known complication of metallic implants due to deposition of metallic parts and building up in the soft tissues [58]. Metallosis has been shown to affect nearby neural structures by causing granuloma/pseudotumor formation with mechanical compression [59–64].

In SAM images, the AI increased as the distance between the metallic body and the tissue sample decreased, which is evidence of Pb mobilization from a gunshot. Heavy metals accumulated in the tissue increases the elasticity, therefore, the AI of the tissue. In the tissue close to the gunshot, the AI was measured to be higher than 2 MRayl almost everywhere, while, in the tissue away from the gunshot, the AI was greater than 2 MRayl only at certain locations. SEM images demonstrate that the tissue obtained far away from the gunshot is found to keep its original structure, whereas the tissue obtained near to the gunshot is deformed and torn up. EDS and ICP-OES results show that Pb, Fe, Cr, and Mn levels are found to be higher in the region close to the gunshot when compared to the distant region. The differences in elemental composition in both regions can also play a role in changing the morphology of tissues. Elements measured in blood and serum do not express an apparent residue mobilization since it has been found that during the first 6 months after a trauma, there was a tendency of blood Pb level elevation and then stabilization after that period [7]. In the literature, we came across no data on the relationship between trace and toxic elements in the gluteal area with one metallic pellet close to the sciatic nerve and our results pointed out that Pb mobilizes from the gunshot into the tissues of the critical organs and changes the concentrations of elements such as Fe, Cr, Mn, and Zn in these organs.

Our findings indicate that tissues closest to metallic bullets suffer from local metallosis. We cannot be absolutely sure about the leading cause of neuropathy in our patient; however, no improvement after the first surgery for lumbar disc herniation indicated another factor. She had local tenderness in the deep gluteal area, which is a sign of sciatic nerve injury. Another possibility was that she suffered from “double hit” where sciatic nerve injury compression aggravated each other. We did not try to obtain a biopsy from the sciatic nerve since it causes damage to a nerve with subsequent neurological deterioration.

## Conclusion

In this study, we present the capability of SAM, SEM, EDS, and ICP-OES in determining the metal toxicity in tissues due to a gunshot injury for the first time. SAM maps of the tissues show morphological and chemical differences at differing distances to the metallic body. SEM and EDS results confirm SAM results by obtaining dissimilar images and elemental distributions for these tissues. Element analysis by ICP-OES also shows explicitly different element levels within the tissues. Consequently, we can say that SAM is capable of observing metal mobilization from a gunshot into the tissues.
